# Reasons for cigarette and tobacco product use, dual use, and poly use among university students

**DOI:** 10.18332/tid/195379

**Published:** 2024-11-22

**Authors:** Mark J. M. Sullman, Maria E. Gras, Jiawei D. Hughes, Ioulia Papageorgi, Fran Calvo, Sílvia Font-Mayolas

**Affiliations:** 1 Department of Social Sciences, University of Nicosia, Nicosia, Cyprus; 2 Quality of Life Research Institute, Universitat de Girona, Girona, Spain; 3 Department of Creative Arts Therapies, College of Nursing and Health Professions, Drexel University, Philadelphia, United States; 4 School of Humanities and Social Sciences, Department of Social Sciences, University of Nicosia, Nicosia, Cyprus

**Keywords:** polytobacco use, reasons, e-cigarettes, waterpipe, young adults

## Abstract

**INTRODUCTION:**

Tobacco use remains a significant public health issue worldwide, causing over 7 million deaths annually. Polytobacco use has become a common phenomenon. This study aims to analyze reasons for cigarette and tobacco product use, dual use and poly use among university students by sex, in China and Cyprus, selected owing to their still high consumption rates.

**METHODS:**

An online survey was conducted among 589 university students (55% women) with a mean age of 24.2 years from Guangzhou, China, and the Republic of Cyprus. Participants reported their use of cigarettes, e-cigarettes, and waterpipes, as well as their reasons for using these products. Statistical analyses included chi-squared tests, and effect sizes were computed using the phi (φ) coefficient.

**RESULTS:**

The Chinese participants gave as reasons for using cigarettes: ‘because of the taste’, ‘because it looks cool’, ‘to get high’ and ‘because friends and family use them’. These were less frequently given by the Cypriots who opted more for ‘to relax and relieve tension’. Compared to the Cypriot participants, the more frequent reasons given for consuming e-cigarettes and waterpipes among Chinese students were: ‘due to boredom’, ‘because it looks cool’, ‘to get high’, ‘because I am hooked’, ‘because friends or family members use them’, ‘because they are less harmful than regular cigarettes’ and ‘because friends of family members allow their use more’. Dual or poly users felt more addicted than those who practiced single use. Among e-cigarette users, dual users and poly users valued the taste of the product more than single users. In general, men found more reasons for tobacco use than women did.

**CONCLUSIONS:**

This study shows substantial differences in the motivation for using these products according to the product type, number of tobacco/nicotine products used, country, and sex. Understanding the reasons for tobacco use can inform targeted interventions aimed at reducing tobacco consumption among young adults.

## INTRODUCTION

Tobacco use is one of the leading causes of death and illness worldwide, with more than 7 million people dying due to tobacco use each year^1,2^. Although the global prevalence of tobacco use has decreased since 1990 in both men and women, the growth of the world population has led to an increase in the number of users^3^. For instance, in countries such as China, over half of the men continue to smoke^3,4^, and in Cyprus, the prevalence of cigarette smoking is among the highest in the EU^5^. The tobacco control policies, cultural attitudes, and social norms in each country may influence these differences. Nevertheless, understanding the patterns and motivations for tobacco use in each country is crucial for developing effective public health interventions.

University students represent a critical population for studying tobacco use. This life stage is often marked by experimentation and the formation of long-term habits, making it an important period for intervention^6,7^. Previous research has shown high rates of tobacco use among university students. Still, there is a need for more detailed studies that explore the reasons behind their use of different tobacco products^6,7^.

Historically, conventional cigarettes dominated the tobacco market. However, in recent years, tobacco products such as e-cigarettes, heated tobacco, and flavored tobacco have gained prominence, along with the reappearance of waterpipes, also known as shisha or hookah^8^. Adolescence and young adulthood are the main periods of experimentation with these tobacco products, which can be the gateway to regular nicotine use^9-11^.

Among young adults, a group that has received attention in previous studies are university students, who usually have high tobacco use rates and where effective interventions for tobacco use cessation are still inconclusive^12^. Among a sample of college students in China, cigarettes (7.8%) and waterpipes (23.8%) were the most common forms of tobacco use, with cigarette consumption more common among men (15% vs 1.1% women) and waterpipe use more common among women (67% vs 33% men)^13^. In Cyprus, smoking rates among young adults (15–24 years) have been reported to be as high as 28%, and among Turkish university students, 38.4% reported current/ever waterpipe use^14^. Similar to the sample of Chinese students, waterpipe use was higher among women (70.9%) than men (29.1%). However, fewer students from China have reported trying e-cigarettes (8.2%) or daily use (1.1%)^15^.

Similar to the common phenomenon of polydrug use in drug consumption, polytobacco use is also prevalent in the case of nicotine^16^. Polytobacco use was initially defined as the current use of cigarettes and one or more tobacco products^17^. Researchers subsequently came to distinguish between dual consumption (current use of two or more nicotine or tobacco products) and polytobacco use (current use of three or more nicotine or tobacco products)^18-21^. Studies with Chinese university students found that 16.3% of men and 2.2% of women were current cigarette users, 6.6% of men and 2.7% of women were current e-cigarette users, 20.9% of men and 5.3% of women were current dual users, and 51.2% of the dual users had started as cigarette-only users^22^.

Understanding the reasons why young people use nicotine and tobacco products has been the aim of several previous studies in different countries^23-25^. For example, in a study of Chinese university students, the main factors motivating e-cigarette use, especially in men, were the belief that e-cigarettes were less harmful than cigarettes or not harmful at all, and that they helped them to quit smoking^22^. Among Turkish university students, waterpipe smokers perceived waterpipe use as less addictive than non-waterpipe tobacco users and considered waterpipe use as helpful for relaxation^16^. Thus, reasons for smoking vary in different regions of the world and also according to sex^26,27^. However, few studies have examined the reasons for dual use and the poly use of nicotine and tobacco products by sex in countries with still high consumption rates, such as China and Cyprus.

The main objective of the present study was to analyze reasons for cigarette and tobacco product use by type of substance used (cigarette, e-cigarette, waterpipe) among a sample of university students from China and Cyprus. The secondary objectives were to investigate reasons for tobacco use (mono/single/exclusive users, dual users, poly users) by country and sex.

## METHODS

### Study design and participants

This cross-sectional online survey was based on previous research on nicotine use^28^. It was conducted using snowball sampling at Guangzhou University (Republic of China) and at the University of Nicosia (Republic of Cyprus) during November and December 2019. A convenience sample of 589 university students completed the survey.

Participation was voluntary and without any form of inducement. The inclusion criteria were: aged ≥18 years, ability to read and understand the local language (Mandarin in China, and Greek or English in Cyprus), and attending a university in the province of Guangzhou in China, and in Cyprus. The online survey was shared through a campus network at each university. Participants were self-selected for the study, were informed of their rights as participants, and provided informed consent. A snowball sampling approach was also used, as students were asked to forward the survey to other university students studying in Guangzhou and Cyprus. This study was approved by the Social Science Ethics Research Board (SSERB 0053) at the University of Nicosia, Cyprus.

### Measures

*Demographics*

Respondents reported their gender (men/women), age (years), and ethnicity (Chinese, Greek Cypriot, Greek, other European, and Turkish Cypriot).

*Cigarette and tobacco products use*

Participants responded to the question: ‘How frequently have you smoked regular cigarettes during the past 30 days?’. The same question was adapted to e-cigarettes and waterpipes. Response options were ‘never’, ‘occasionally’, ‘once a week’, ‘more than once a week, but not every day’, and ‘every day’. Participants who reported ‘never’ using tobacco were classified as non-users, and those who answered ‘occasionally’, ‘once a week’, ‘more than once a week, but not every day’, or ‘every day’ were classified as current users.

*Reasons for smoking/vaping*

The reasons for tobacco use were based on previous research in this area^23,24^. Students who reported having vaped or smoked at some time were asked: ‘What were your main reasons for using regular cigarettes/e-cigarettes/waterpipes?’. There were nine possible reasons given: ‘to experiment, to see what it is like’, ‘because it tastes good’, ‘because of boredom, nothing else to do’, ‘to have a good time with my friends’, ‘to relax or relieve tension’, ‘because it looks cool’, ‘to get high’, ‘because I am hooked - I have to have it’, ‘because friends or family members use them’, and ‘because e-cigarettes/waterpipes without nicotine are less harmful than regular cigarettes’. Participants who reported having used e-cigarettes or waterpipes were asked for three more reasons, which included: ‘to help me quit regular cigarettes’, ‘because e-cigarettes/waterpipes with nicotine are less harmful than regular cigarettes’, and ‘because friends or family members permitted e-cigarettes/waterpipes more than regular cigarettes’. Response options were: ‘definitely yes’, ‘probably yes’, ‘probably no’, and ‘definitely no’. Students could select multiple reasons.

### Statistical analysis

Chi-squared tests were used to compare percentages. Effect sizes were computed using the phi (φ) coefficient. For the data analysis, the categories ‘definitively yes’ and ‘probably yes’ were merged, and ‘definitively no’ and ‘probably no’ were combined. The level of significance was set at 0.05, and all tests were two-tailed. Paired samples were compared using the Cochran Q test. All analyses were performed using SPSS v23 (IBM Corp, Armonk, NY, USA).

## RESULTS

The sample was composed of 589 participants (55% women), with a mean age of 24.2 years (SD=5.3). Of the participants, 47.7% (n=281) were from China (ethnicity: all Chinese) and 52.3% (n=308) were from Cyprus (ethnicity: Greek Cypriot 45.8%, Greek 11.4%, other European 16.2%, Turkish Cypriot 1.3%, and other 25.3%). With regard to gender, 53.7% of the participants from China were men (n=151) and 46.3% women (n=130), while the figures for Cyprus were 37% men (n=114) and 63% women (n=194).

### Cigarette and tobacco products use

Overall, 48.6% (n=286) were current cigarette users, 33.3% (n=196) were e-cigarette users, and 52.3% (n=308) were waterpipe users. By country, nicotine use was higher in Cyprus (59.1%, 36.7%, and 74%, respectively) than in China (37%, 29.5%, and 28.5%, respectively), with significant differences in cigarette use (χ^2^=28.7; p<0.001; φ=0.22) and waterpipe use (χ^2^=122.2; p<0.001; φ=0.42).

By sex, cigarette, e-cigarette, and waterpipe use were higher in men (55.1%, 43.4%, and 54%, respectively) than in women (43.2%, 25%, and 50.9%, respectively), but differences were only significant for cigarette use (χ^2^=8.2; p=0.004; φ=0.12) and e-cigarette use (χ^2^=22.2; p<0.001; φ=0.19), not for waterpipe use (χ^2^=0.54; p=0.46).

### Cigarette and tobacco products use, dual use, and poly use, by sex

Over one-third (34.3%, n=208) of the participants defined themselves as non-users of regular cigarettes, electronic cigarettes, or waterpipes. By country, non-users represented 54.9% in China (n=154) and only 17.5% in Cyprus (n=54) (χ^2^=32.5; p<0.001; φ=0.23). Amongst tobacco users, (n=381; China, n=127; Cyprus, n=254), 29.2% of the participants reported mono use, 37.5% reported dual use, and 33.3% reported poly use. Table 1 shows the percentage of participants according to cigarette and tobacco product use, country, and sex. Significant differences were found between men and women in Cyprus, with more men than women being poly users and more women than men being dual users (χ^2^=13.9; p<0.001; φ=0.23), but no differences were found by sex in China (χ^2^=1.9; p=0.39). If we compare tobacco product use amongst men in both countries, there were no significant differences (χ^2^=3.8; p=0.15), and the same was true of women (χ^2^=5.3; p=0.07).

**Table 1:** Cigarette and tobacco products use among university students from Guangzhou, China, and the Republic of Cyprus, by sex (N=387)

*Cigarette and tobacco products use*	*China*		*Cyprus*	
	*Men* *% (n)*	*Women* *% (n)*	*Men* *(%) n*	*Women* *% (n)*
Mono use	30.0 (27)	39.5 (17)	21.4 (21)	30.8 (48)
Dual use	36.7 (33)	25.6 (11)	31.6 (31)*	44.9 (70)*
Poly use	33.3 (30)	34.9 (15)	46.9 (46)*	24.4 (38)*

* Significant differences between men and women. Chi-squared test was used to compare by country and by sex.

Among mono users, 38.1% (n=43) were only cigarette users (China: 56.8%, n=25; Cyprus: 26.1%, n=18), 9.7% (n=11) were only e-cigarette users (China: 20.5%, n=9; Cyprus: 2.9%, n=2), and 52.2% (n=59) were only waterpipe users (China: 22.7%, n=10; Cyprus: 71.0%, n=49). By gender, 50% of men and 29.2% of women were only cigarette users, 14.5% of men and 6.2% of women were only e-cigarette users, and 35.4% of men and 64.6% of women were only waterpipe users.

Table 2 shows the percentage of dual users according to substance use, country, and sex. Although in China, more men than women combined waterpipe with cigarette or e-cigarette use and more women than men combined cigarettes and e-cigarettes, differences were not significant (χ^2^=0.8; p=0.76). As for Cyprus, more women than men combined cigarettes with waterpipes or e-cigarettes, and more men than women combined waterpipes and e-cigarettes. However, the differences were again not significant (χ^2^=3.8; p=0.19).

**Table 2:** Dual users, according to the substances consumed among university students from Guangzhou, China, and the Republic of Cyprus, by sex (N=145)

*Substances*	*China*		*Cyprus*	
	*Men* *(N=33)* *% (n)*	*Women* *(N=11)* *% (n)*	*Men* *(N=31)* *% (n)*	*Women* *(N=70)* *% (n)*
Waterpipe and cigarettes	36.4 (12)	27.3 (3)	64.5 (20)	77.1 (54)
Waterpipe and e-cigarettes	24.2 (8)	18.2 (2)	32.3 (10)	15.7 (11)
Cigarettes and e-cigarettes	39.4 (13)	54.5 (6)	3.2 (1)	7.1 (5)

Chi-squared test was used to compare by country and by sex.

### Reasons for smoking among cigarette, e-cigarette, and waterpipe users

For the whole sample, among regular cigarette users, the most (75.7%) reported reason was ‘to relax and relieve tension’, followed by ‘to experiment, to see what it is like’ (60.6%). Among e-cigarette users, the most common (75%) reason was also ‘to experiment, to see what it is like’, followed by ‘because it tastes good’ (68%). Among waterpipe users, the most commonly provided reason was ‘to have a good time with my friends’ (78.8%), followed by ‘to experiment, to see what it is like’ (77.7%), and by ‘because it tastes good’ (77.7%).

Tables 3 and 4 show the reasons for smoking/vaping by cigarette and tobacco product use and the results of the Cochran Q test for paired samples, for the China and Cyprus participants, respectively. In the China sample, the reasons ‘because it tastes good’ and ‘because friends or family members allowed me to use e-cigarettes more than regular cigarettes’ were significantly more frequent in relation to the e-cigarette. However, no differences were found by substance in the other reasons. In the Cyprus sample, the reason ‘to experiment, to see what it is like’ was reported significantly more in the case of e-cigarettes and waterpipes. In contrast, the reason ‘because it tastes good’ was reported more frequently in the case of waterpipes, followed by e-cigarettes. ‘To have a good time with my friends’ was reported more frequently for waterpipe use, followed by cigarettes, but ‘to relax or relieve tension’ was mainly reported in reference to cigarettes. The reason ‘to get high’ was significantly less frequent for e-cigarettes than the other substances and ‘because I am hooked’ was more frequently reported in reference to the use of cigarettes. E-cigarette use was more frequent than waterpipe use because ‘they are less harmful than regular cigarettes’ and ‘because friends or family allowed me to use them more than regular cigarettes’.

**Table 3:** Reasons for smoking/vaping (percentage of ‘probably yes’ or ‘definitely yes’ responses) by cigarette, e-cigarette, and waterpipe use, with the Cochran Q test results for paired samples among university students from Guangzhou, China

*Reasons*	Cigarettes* *% (n)*	E-cigarettes* *% (n)*	Waterpipe* *% (n)*	*Q (p)*
1. To experiment, to see what it is like (N=41)	63.4 (26)	73.2 (30)	80.5 (33)	3.2 (0.20)
2. Because it tastes good (N=41)	48.7 (20)	75.6 (31)*	58.5 (24)	7.15 (0.03)
3. Because of boredom, nothing else to do (N=41)	53.7 (22)	63.4 (26)	63.4 (26)	1.28 (0.53)
4. To have a good time with my friends (N=41)	53.7 (22)	41.5 (17)	65.9 (27)	6.0 (0.05)
5. To relax or relieve tension (N=40)	70.0 (28)	55.0 (22)	55.0 (22)	4.0 (0.14)
6. Because it looks cool (N=41)	46.3 (19)	53.7 (22)	60.9 (25)	2.7 (0.26)
7. To get high (N=40)	40.0 (16)	32.5 (13)	32.5 (13)	1.2 (0.55)
8. Because I am hooked. I have to have it (N=40)	52.5 (21)	40.0 (16)	35.0 (14)	4.1 (0.13)
9. Because friends or family members use them (N=40)	55.0 (22)	52.5 (21)	47.5 (19)	0.8 (0.68)
10. To help me quit regular cigarettes (N=52)	-	59.6 (31)	44.2 (23)	3.2 (0.07)
11. Because e-cigarettes/waterpipe without nicotine are less harmful than regular cigarettes (N=51)	-	54.9 (28)	45.1 (23)	1.7 (0.20)
12. Because friends or family members allowed e-cigarettes/waterpipe more than regular cigarettes (N=40)	-	72.5 (29)*	57.5 (23)	4.5 (0.03)

* Significant differences between pairs.

**Table 4:** Reasons for smoking/vaping (percentage of ‘probably yes’ or ‘definitely yes’ responses) by cigarette, e-cigarette, and waterpipe use, with the Cochran Q test results for paired samples among university students from the Republic of Cyprus

*Reasons*	Cigarettes* *% (n)*	E-cigarettes* *% (n)*	Waterpipe* *% (n)*	*Q (p)*
1. To experiment, to see what it is like (N=70)	60.0 (42)*	75.7 (53)	78.6 (55)	11.3 (0.004)
2. Because it tastes good (N=68)	22.1 (15)*	76.5 (52)**	92.6 (63)	72.9 (<0.001)
3. Because of boredom, nothing else to do (N=68)	45.6 (31)	47.1 (32)	42.6 (29)	0.5 (0.80)
4. To have a good time with my friends (N=70)	58.6 (41)*	40.0 (28)**	87.1 (61)***	43.6 (<0.001)
5. To relax or relieve tension (N=68)	82.3 (56)*	54.4 (37)	44.1 (30)	28.6 (<0.001)
6. Because it looks cool (N=67)	25.4 (17)	20.9 (14)	31.3 (21)	3.9 (0.14)
7. To get high (N=60)	23.3 (14)	8.3 (5)*	21.7 (13)	8.6 (0.01)
8. Because I am hooked. I have to have it (N=61)	47.5 (29)*	13.1 (8)	6.6 (4)	36.1 (<0.001)
9. Because friends or family members use them (N=61)	34.4 (21)	24.5 (15)	34.4 (21)	3.6 (0.17)
10. To help me quit regular cigarettes (N=85)	-	41.2 (35)	15.3 (13)	17.3 (<0.001)
11. Because e-cigarettes/waterpipe without nicotine are less harmful than regular cigarettes (N=83)	-	39.8 (33)*	27.7 (23)	4.2 (0.04)
12. Because friends or family members allowed e-cigarettes/waterpipe more than regular cigarettes (N=83)	-	12.0 (10)	12.0 (10)	0 (1)

* Indicate significant differences between pairs.

### Reasons for cigarette, e-cigarette, and waterpipe use by polytobacco status

Among cigarette users, there were no significant differences in the reasons for tobacco use by polytobacco status, except for the reason ‘Because I am hooked’: both dual users (52.4%) and poly users (50.4%) gave this answer than single users (38.9%) (χ^2^=7.6; p=0.02; φ=0.17).

Among e-cigarette users, there were no significant differences in the answers to the reasons for use by polytobacco status, except for ‘because it tastes good’; 81.4% of poly users, 64.7% of dual users, and only 48% of mono e-cigarette users answered yes or probably yes (χ^2^=11.9; p=0.003; φ=0.26).

Among waterpipe users, differences were only significant for ‘because it looks cool’. Significantly more poly users (43.9%) and dual users (33%) answered yes or probably yes, than did waterpipeonly users (24.5%) (χ^2^=6.5; p=0.04; φ=0.15).

### Reasons for cigarette, e-cigarette, and waterpipe use by country and sex

**Figure 1:**
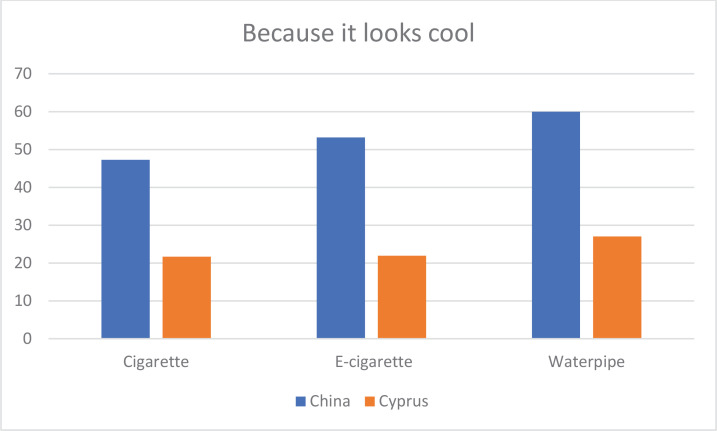
Percentages of ‘probably yes’ or ‘definitely yes’ responses to the reason ‘because it looks cool’ for smoking/vaping each substance among university students from Guangzhou, China, and the Republic of Cyprus

Table 5 shows the percentages for the answers ‘probably yes’ or ‘definitely yes’ to the reasons for smoking/vaping each substance by country. Significantly more participants from China used cigarettes ‘because it tastes good’, ‘because it looks cool’, ‘to get high’ and ‘because friends and family use them’, than participants from Cyprus. The difference in the reason for taste is illustrated in Figure 1. In contrast, the participants from Cyprus gave the reason ‘to relax and relieve tension’ more frequently than those from China. The participants from China reported that they used e-cigarettes ‘because of boredom’, ‘because it looks cool’, ‘to get high’, ‘because I am hooked’, ‘because friends or family members use them’, ‘to help me quit regular cigarettes’, ‘because they are less harmful than regular cigarettes’ and ‘because friends or family members allow me to use them more’, more frequently than those from Cyprus. They also reported that they used waterpipes ‘because of boredom’, ‘because it looks cool’, ‘because I am hooked’, ‘to help me quit regular cigarettes’, ‘because they are less harmful than regular cigarettes’ and ‘because friends or family members allow me to use them more’, more frequently than those from China.

**Table 5:** Reasons for smoking/vaping (percentage of ‘probably yes’ or ‘definitely yes’ responses) for each substance among university students from Guangzhou, China, and the Republic of Cyprus, by country and the results of a chi-square test

*Reasons*	*Cigarettes*			*E-cigarettes*			*Waterpipe*		
	*China* *% (n)*	*Cyprus* *% (n)*	*χ^2^ (p)* *φ*	*China* *% (n)*	*Cyprus* *% (n)*	*χ^2^ (p)* *φ*	*China* *% (n)*	*Cyprus* *% (n)*	*χ^2^ (p)* *φ*
1. To experiment, to see what it is like	62.8 (59)	60.8 (101)	0.1 (0.76) -	75.6 (59)	75.0 (78)	0.01 (0.92) -	80.0 (60)	78.7 (166)	0.06 (0.81) -
2. Because it tastes good	46.3 (44)	30.5 (51)	6.5 (0.01) 0.16	67.5 (52)	76.0 (79)	1.6 (0.21) -	56.0 (42)	88.8 (191)	37.9 (<0.001) 0.34
3. Because of boredom, nothing else to do	50.0 (48)	51.5 (88)	0.1 (0.82) -	66.7 (52)	51.0 (53)	4.5 (0.03) 0.16	59.7 (46)	45.0 (94)	4.9 (0.03) 0.13
4. To have a good time with my friends	59.8 (58)	60.5 (42)	0.01 (0.91) -	48.7 (38)	44.0 (44)	0.4 (0.53) -	68.0 (51)	88.0 (190)	15.6 (<0.001) 0.23
5. To relax or relieve tension	69.5 (66)	83.8 (145)	7.5 (0.01) 0.16	61.0 (47)	56.6 (56)	0.4 (0.55) -	56.0 (42)	50.2 (103)	0.73 (0.39) -
6. Because it looks cool	47.3 (44)	21.7 (36)	18.3 (<0.001) 0.26	53.2 (41)	21.9 (21)	18.3 (<0.001) 0.31	60.0 (45)	27.0 (55)	26.0 (<0.001) 0.29
7. To get high	44.7 (42)	18.9 (30)	19.3 (<0.001) 0.27	41.3 (31)	10.2 (9)	21.2 (<0.001) 0.34	30.6 (22)	20.9 (40)	2.7 (0.10) -
8. Because I am hooked. I have to have it	48.9 (46)	47.2 (77)	0.1 (0.79) -	43.4 (33)	16.1 (15)	15.3 (<0.001) 0.29	33.8 (24)	10.1 (19)	20.9 (<0.001) 0.27
9. Because friends or family members use them	50.5 (48)	31.8 (50)	8.7 (0.003) 0.18	46.1 (35)	24.7 (22)	8.2 (0.004) 0.22	43.1 (31)	30.9 (60)	3.4 (0.06) -
10. To help me quit regular cigarettes	-	-	-	55.8 (43)	39.6 (38)	4.5 (0.03) 0.16	43.2 (32)	13.8 (26)	26.9 (<0.001) 0.31
11. Because e-cigarettes/waterpipe without nicotine are less harmful than regular cigarettes	-	-	-	53.3 (40)	35.1 (33)	5.6 (0.02) 0.18	46.6 (34)	21.3 (42)	16.8 (<0.001) 0.24
12. Because friends or family members allowed e-cigarettes/waterpipe more than regular cigarettes	-	-	-	72.6 (45)	12.8 (12)	57.6 (<0.001) 0.52	51.4 (37)	15.5 (30)	35.7 (<0.001) 0.34

Significantly more men than women reported using these substances ‘because it looks cool’ (χ^2^=9.3; p=0.002; φ=0.17). Furthermore, significantly more men used cigarettes and e-cigarettes ‘to get high’ than women (cigarettes: χ^2^=9.2; p=0.002; φ=0.17; and e-cigarettes: χ^2^=8.4; p=0.004; φ=0.18). Additionally, more men than women used e-cigarettes ‘because friends or family members allowed these cigarettes more than regular cigarettes’ (χ^2^=7.85; p=0.005; φ=0.18) and used waterpipes ‘because I am hooked’ (χ^2^=4.4; p=0.04; φ=0.12). Conversely, significantly more women than men used waterpipes ‘because it tastes good’. There were no further significant differences by sex.

## DISCUSSION

Our survey indicates that the reasons for tobacco use varied by product type. Regular cigarette users primarily cited relaxation and tension relief as their main motivations, while e-cigarette and waterpipe users were more likely to report the desire to experiment and that they enjoyed the taste. E-cigarette users also believed that e-cigarette use could help them to quit conventional cigarettes. These findings align with previous studies that identified similar motivations for tobacco use among young adults^16,29,30^. These results highlight the need to promote research on new flavors, such as ice flavors or synthetic coolants, and their effects on promoting vaping and dependence^31^. Among waterpipe users, additional reasons commonly reported included wanting to look cool and enjoying time with friends, which may explain the increasing adoption of this method of consumption among young people.

Furthermore, our research reveals differences by country in assessing reasons for tobacco use. In the case of cigarettes, the Chinese participants reported the reasons ‘because it tastes good’, ‘because it looks cool’, ‘to get high’ and ‘because friends and family use them’ more frequently than Cypriots. At the same time, the latter opted for ‘to relax and relieve tension’ more. Compared to the Cypriots, the Chinese participants responded more frequently with the following reasons for consuming e-cigarettes and waterpipes: ‘because of boredom’, ‘because it looks cool’, ‘to get high’, ‘because I am hooked’, ‘because friends or family members use them’, ‘because they are less harmful than regular cigarettes’ and ‘because friends of family members allow me to use them more’. In other words, among the reasons presented for consuming tobacco, the Chinese participants perceived more advantages, such as emotional management, social image, and the usefulness of tobacco products in abandoning conventional cigarettes. These differences highlight the need to continue studying the specific characteristics of smokers by country^27^ and to be attentive to the high number of consumption reasons observed among Chinese smokers^4,5^. Furthermore, these results highlight the need to continue promoting interventions that are considered promising but have limited evidence, particularly those focused on enhancing social-emotional skills^32^.

The analysis of motivations for tobacco use based on the number of products used among cigarette users indicated that those who practiced dual use or poly use, felt more addicted than those who practiced single use. In other words, the more consumption routes, the greater the perception of dependence. Among e-cigarette consumers, dual users and poly users valued the taste of the product more than single users, suggesting that these users were especially susceptible to the pleasurable effects of different tobacco flavors. Among waterpipe users, the greater the number of products used, the more important was the motivation to look cool. The significant differences in motivations between product types are in line with previous research^33^ and highlighted the need for tailored interventions that addressed the specific reasons for use.

The analysis of motivations for tobacco use by sex showed that men, compared to women, used cigarettes, e-cigarettes, and waterpipes because they looked cool, used e-cigarettes and waterpipes to get high, and used e-cigarettes because friends or family members more commonly accepted them than regular cigarettes. Conversely, women, compared to men, used waterpipes for their taste. Therefore, in general, men found more reasons for tobacco use than women did, in line with previous studies in diverse countries^27^. This could explain the sex difference in the prevalence of smoking, although it could also indicate the need to adjust the list of possible reasons by sex. In the latter case, qualitative studies should be used to understand women’s reasons for tobacco use more clearly.

Additionally, our study provides information on the characteristics of tobacco consumption in the samples studied. Men more commonly used cigarettes and e-cigarettes than women. These data are in line with previous research among university students, which also found a higher prevalence of cigarette and e-cigarette consumption in men than in women^13,17^. Nevertheless, no statistically significant differences were found in current waterpipe use between men and women in the present sample. These waterpipe use rates are higher than those previously reported for men and lower than those for women in studies of university students, which also noted significant sex differences^16^. These findings underscore the importance of promoting preventive smoking interventions on university campuses, as suggested by Xie et al.^14^, with a comprehensive approach to various forms of tobacco use.

### Implications for interventions

Understanding the motivations behind tobacco use is crucial for developing effective clinical interventions and refining public health policies regarding cultural and marketing factors^3^. Specifically, the differences detected among Chinese and Cypriot smokers in this study indicate different sensitivities to the possible reasons for consumption. Therefore, there is a need to incorporate these differences within national prevention programs and in emerging forms such as waterpipe use^34^. Gender-sensitive messages^27^ may be necessary to effectively target the different motivations and usage patterns observed in men and women, and in the case of China, to discourage smoking initiation in young women^4^. The low scores among women for the consumption reasons offered in the study in comparison to men highlight the need to delve into the reasons why women use tobacco both at a clinical level – to offer appropriate levels of counseling^35^ – and in preparation for future preventive campaigns. The different reasons for consumption detected according to the type and number of substances used also indicate the need to be sensitive to specific motivations when caring for mono users, dual users, and poly users.

### Strengths and limitations

This study has several strengths, such as including young people from two very different countries (China and Cyprus). There has also been very little scientific literature on tobacco use that has included participants from different countries. The study also focuses on multiple tobacco products (cigarettes, e-cigarettes, and waterpipes), which provides a more comprehensive understanding of tobacco consumption patterns among young adults. The study’s detailed analysis of motivations for tobacco use, including differences by sex and type of product, offers valuable insights for developing tailored interventions.

However, this work also has several limitations. Using a convenience sample may introduce bias and limit the generalizability of the findings. Furthermore, the survey relied on self-reporting, which could be susceptible to potential weaknesses such as social desirability bias. Furthermore, apart from conventional cigarettes, only two other tobacco products were included. A further limitation is that the data are not generalizable to other countries. Also, the study’s cross-sectional design does not allow for causal inferences. Future research should consider longitudinal designs to understand the dynamics of tobacco use over time more accurately.

## CONCLUSIONS

This study highlights significant differences in reasons for smoking among university students by country and sex. The findings underscore the importance of understanding the specific motivations for tobacco use in different countries, which can be used to inform targeted interventions. Addressing the diverse reasons for tobacco and polytobacco consumption and considering gender differences, can enhance the effectiveness of public health strategies aimed at reducing tobacco use among young adults.

## Data Availability

The data supporting this research are available from the authors on reasonable request.
